# Real-world effectiveness of SGLT2 inhibitors in patients with HF and ESKD: a multicenter cohort study

**DOI:** 10.3389/fcvm.2026.1652863

**Published:** 2026-01-16

**Authors:** Jheng-Yan Wu, Kuan-Jui Tseng, Chia-Li Kao, Kuo-Chuan Hung, Tsung Yu, Yu-Min Lin

**Affiliations:** 1 Department of Nutrition, Chi Mei Medical Center, Tainan, Taiwan; 2 Department of Public Health, College of Medicine, National Cheng Kung University, Tainan, Taiwan; 3 Division of Cardiology, Department of Internal Medicine, Chi Mei Medical Center, Tainan, Taiwan; 4 Department of Anesthesiology, E-Da Hospital, I-Shou University, Kaohsiung City, Taiwan; 5 Department of Anesthesiology, Chi Mei Medical Center, Tainan, Taiwan; 6 Division of Cardiology, Department of Internal Medicine, Chi Mei Medical Center, Chiali, Tainan, Taiwan

**Keywords:** end-stage kidney disease, heart failure, major adverse cardiovascular events, major adverse kidney events, sodium-glucose cotransporter-2 inhibitors

## Abstract

**Background:**

Patients with heart failure (HF) and end-stage kidney disease (ESKD) face a high burden of mortality and hospitalization, yet effective therapeutic options remain limited. While sodium-glucose cotransporter-2 inhibitors (SGLT2i) have demonstrated cardiovascular and renal benefits across various chronic kidney disease (CKD) stages, their effects in patients with HF and ESKD remain uncertain due to their exclusion from major clinical trials.

**Objectives:**

This study aimed to evaluate the effectiveness of SGLT2i in reducing one-year clinical outcomes, including all-cause hospitalization, all-cause mortality, and acute pulmonary edema in patients with HF and ESKD.

**Methods:**

A retrospective cohort study was conducted using the TriNetX network. Patients with HF and ESKD diagnosed between 2016 and 2025 were identified and categorized into SGLT2i users and non-users. The primary outcome was a composite of all-cause hospitalization, all-cause mortality, and acute pulmonary edema at 1 year. Secondary outcomes included each component of the primary composite. Propensity score matching (PSM) was applied to balance baseline characteristics. Cox proportional hazard models estimated hazard ratios (HRs) with 95% confidence intervals (CIs) for primary and secondary outcomes. Sensitivity analyses, including negative control and landmark analyses, were performed to validate findings.

**Results:**

After PSM, 10,032 patients were analyzed. SGLT2i use was associated with a significantly lower risk of the primary composite outcome (HR 0.80, 95% CI 0.76–0.84, *p* < 0.001). Subgroup analyses demonstrated consistent benefits across different HF phenotypes, comorbidities, and different types of SGLT2i.

**Conclusions:**

SGLT2i are significantly associated with reduced 1 year all-cause hospitalization, mortality, and acute pulmonary edema in HF and ESKD patients. These findings highlight the potential therapeutic role of SGLT2i in this high-risk population, warranting further clinical trials to confirm their efficacy and safety.

## Introduction

In the United States, the prevalence of cardiovascular disease among patients with chronic kidney disease (CKD) is as high as 63%, compared to just 5.8% in individuals without CKD. Moreover, this prevalence increases in direct proportion to CKD severity (1). In patients with dialysis-dependent end-stage kidney disease (ESKD), the risk of cardiovascular mortality is 20 times higher than that of control subjects without CKD (2). Heart failure (HF) is the most common cardiovascular complication in patients with chronic kidney disease (CKD), with its prevalence rising as kidney function declines (3). In dialysis patients, HF at the initiation of dialysis serves as a strong and independent predictor of both short-term and long-term mortality in individuals undergoing hemodialysis (HD) (4) and peritoneal dialysis (PD) (5).

Fortunately, sodium-glucose transporter type 2 inhibitors (SGLT2i) have emerged as the new standard care for CKD and HF therapy due to their distinct renal and cardiovascular protective effects (6–8). However, in clinical practice, SGLT2i are discontinued in patients with ESKD due to the lack of safety data, as these patients were historically excluded from major clinical trials (9, 10). Interestingly, a recent systematic review and meta-analysis demonstrated that SGLT2i therapy reduces the risk of renal and HF outcomes across a broad range of estimated glomerular filtration rate (eGFR) categories, with the greatest relative risk reduction observed in patients with reduced kidney function. However, it should be noted that participants with eGFR <20 mL/min/1.73 m^2^ and those on dialysis were not included in these trials (11). This result might suggest that the renal and cardiovascular protective effects of SGLT2i operate through distinct pathophysiological mechanisms.

Nevertheless, these trial excluded patients with ESKD, and not all participants were diagnosed with HF. Therefore, whether SGLT2i provides benefits for patients with both ESKD and HF remains unknown. To address this knowledge gap, we conducted an exploratory study using real-world data from the TriNetX database to evaluate the effectiveness of SGLT2i on one-year clinical outcomes in patients with ESKD and HF. In contrast to randomized controlled trials, which often exclude patients with end-stage kidney disease and advanced comorbidities, real-world cohort studies allow evaluation of treatment effectiveness in routine clinical practice. Such evidence may better reflect treatment patterns, concomitant therapies, and outcomes in high-risk populations commonly encountered by clinicians.

## Methods

### Data sources

This retrospective cohort analysis utilized the TriNetX platform, a worldwide federated health research network that aggregates electronic health records from roughly 150 healthcare organizations, encompassing approximately 140 million patients. The platform collects diverse clinical data including diagnoses, procedures, medications, laboratory values, and genetic information. Since TriNetX provides only aggregated statistics and anonymized summaries without accessing protected health information or participating in study-specific activities, it operates under an exemption granted by the Western Institutional Review Board. The research was conducted following the Strengthening the Reporting of Observational Studies in Epidemiology (STROBE) guidelines (12).

### Ethical approval

This study was approved under exemption from the Western Institutional Review Board as it utilized de-identified and aggregated patient data from the TriNetX research network, which does not include protected health information or enable the identification of individual patients. As a result, no additional Institutional Review Board approval or informed consent was required.

### Study design

Participants aged ≥18 years with concurrent diagnosis of HF and ESKD documented between January 2016 and February 2025 were eligible to minimize the time-related biases. HF was identified using International Classification of Diseases, Tenth Revision, Clinical Modification (ICD-10-CM) codes I50, while ESKD was identified using ICD-10-CM code N18.6. Patients with isolated HF or isolated ESKD were excluded, and only those with both diagnoses were included in the analytical cohort. Within this HF-ESKD cohort, patients were then categorized into two groups. Those who received SGLT2i within one year after the index date formed the SGLT2i group, while those without SGLT2i use were assigned to the control group. The index date was defined as the date of first recorded use of SGLT2i for the SGLT2i group, and the date of concurrent HF and ESKD diagnosis for the control group; the baseline period was the year preceding this index date. Exclusion criteria were as follows: patients younger than 18 years; those without a documented concurrent diagnosis of both HF and ESKD; those lacking baseline demographic or clinical information; and those without any follow-up records after the index date. Detailed coding algorithms for identifying baseline characteristics, clinical diagnoses, procedures, medications, and laboratory parameters appear in Supplementary Table S1.

### Covariates

We matched our statistical models for the following covariates: age, sex, race, left ventricular ejection fraction, comorbidities (diabetes mellitus, metabolic disorders, defined as obesity, dyslipidemia, and metabolic syndrome; see Supplementary Table S2 for ICD-10 codes, hypertension, dyslipidemia, cancer, chronic obstructive pulmonary disease, dementia, prior stroke, and atrial fibrillation), and medication (digitalis glycosides, beta blockers, alpha blockers, calcium channel blockers, furosemide, angiotensin ii inhibitor angiotensin-converting enzyme inhibitors, angiotensin receptor–neprilysin inhibitor, ivabradine, vericiguat, spironolactone, nitrates, statins, ezetimibe, PCSK9 inhibitors [evolocumab or alirocumab], and antiplatelet medications). A full list of covariates and their operational definitions is presented in Supplementary Table S2.

### Outcomes and follow-up

The primary outcome was the composite of all-cause mortality, all-cause hospitalization, and acute pulmonary edema. Secondary outcomes consisted of each component of the primary composite outcome. In addition, we also evaluated safety outcomes, specifically syncope and diabetic ketoacidosis. These outcomes were chosen due to their clinical relevance and potential association with SGLT2i therapy. The outcome follow-up period was the day after the index date and continued until the occurrence of an outcome event, final clinical visit, death, or one year after the index date, whichever came first. All diagnostic, procedural, and visit codes used for identifying the outcomes are described in Supplementary Table S3.

### Statistical analysis

Continuous variables are presented as mean with standard deviation, while categorical variables are shown as counts and percentages. To ensure comparable baseline characteristics between groups before conducting primary, subgroup, and sensitivity analyses, propensity score matching (PSM) was implemented. The propensity scores were calculated using the previously mentioned covariates; balanced covariates were defined as those with an absolute standardized mean difference less than 0.1 (13). Cox proportional hazards models were used to calculate hazard ratios (HRs) with their 95% confidence intervals (CIs). For survival analysis, Kaplan–Meier curves were generated and compared using log-rank tests. All statistical analyses were conducted within the TriNetX platform.

We conducted additional subgroup analyses focusing on diabetes mellitus, obesity, coronary artery disease (CAD), classification of HF, and types of SGLT2i. To validate the robustness of our findings, we evaluated negative control outcomes including traumatic brain injury, and skin cancer, which were chosen because current knowledge does not indicate any expected association between these conditions and the use of SGLT2i. Furthermore, we calculated E-values to assess the potential impact of unmeasured confounding factors (14). Last, the Landmark analysis was performed to minimize the time-dependent confounding factor (15). Lastly, we varied the time window for SGLT2i initiation to evaluate whether different initiation periods influenced the results. Additionally, we used dipeptidyl peptidase-4 inhibitors (DPP4is) as an active comparator to assess the consistency of SGLT2i effectiveness. To further ensure the robustness of our findings, we conducted a sensitivity analysis restricted to patients in the SGLT2i group who had a second prescription recorded between 6 months and 1 year after the index date. This analysis aimed to confirm that the observed effects remained consistent among patients with continued SGLT2i use throughout the follow-up period.

## Results

### Patient selection

**Figure 1:**
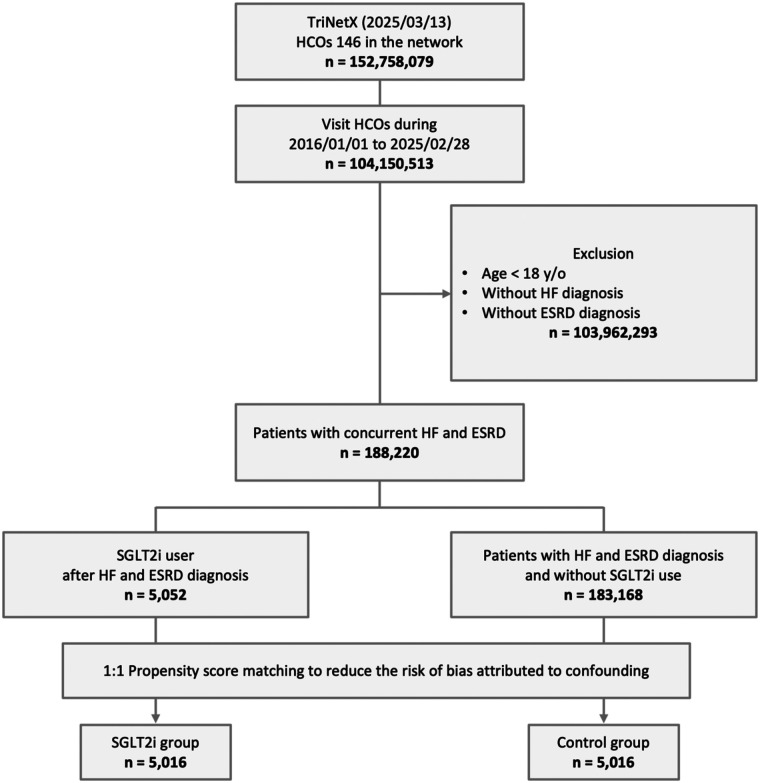
Study cohort process. ESKD, end-stage kidney disease; HCOs, healthcare organizations; HF, heart failure; SGLT2i, sodium-glucose co-transporter 2 inhibitor; y/o, years old.

From an initial cohort of 152,758,079 patients across 146 HCOs within the TriNetX network, 104,150,513 individuals visited between January 1, 2016, and Feburary 28, 2025. Of these, 103,962,293 were excluded for meeting the exclusion criteria. Among the remaining 188,220 patients with concurrent HF and ESKD, 5,052 were treated with SGLT2i, and 183,168 had not used SGLT2i. After 1:1 PSM, 5,016 matched pairs were included in the final analysis (Figure 1). The median follow-up time was 301 days (Q1–Q3: 170–365) in the SGLT2i group and 365 days (Q1–Q3: 258–365) in the control group.

### Demographic characteristics

Before matching, significant differences were observed between the SGLT2i group (*n* = 5,052) and the control group (*n* = 183,149) across multiple demographic and clinical characteristics. The mean age was slightly higher in the SGLT2i group (65.0 ± 12.6 years) compared to the control group (64.2 ± 14.2 years). The proportion of females was lower in the SGLT2i group (34.3%) than in the control group (40.8%). Racial distribution was generally similar between groups, but a slightly higher percentage of Asian patients was observed in the SGLT2i group (10.4% vs. 6.6%). The prevalence of comorbidities, such as diabetes mellitus (77.0% vs. 42.2%), metabolic disorders (88.9% vs. 50.9%), and hypertension (75.8% vs. 47.5%), was markedly higher in the SGLT2i group. Regarding medication use, patients in the SGLT2i group were more likely to be prescribed ACE inhibitors, ARBs, beta blockers, calcium channel blockers, and other cardiovascular drugs. After PSM, the baseline characteristics of the SGLT2i group (*n* = 5,016) and the control group (*n* = 5,016) became more comparable, with all standardized differences reduced to below 0.1 (Table 1).

**Table 1:** Baseline characteristics of SGLT2i and control groups before and after matching.

Variables	Before matching			After matching		
	SGLT2i group (*n* = 5,052)	Control group (*n* = 183,149)	Standardized difference	SGLT2i group (*n* = 5,016)	Control group (*n* = 5,016)	Standardized difference
Age at index, years						
Mean (SD)	65.0 (12.6)	64.2 (14.2)	0.058	65.0 (12.7)	65.3 (13.0)	0.002
Sex, *n* (%)						
Female	1,731 (34.3)	74,682 (40.8)	0.135	1,725 (34.4)	1,765 (35.2)	0.017
Male	3,180 (62.9)	100,867 (55.1)	0.161	3,152 (62.8)	3,121 (62.2)	0.013
Race, *n* (%)						
White	2,211 (43.8)	79,327 (43.3)	0.009	2,204 (43.9)	2,276 (45.4)	0.029
Black or African American	1,445 (28.6)	52,430 (28.6)	0.001	1,432 (28.5)	1,409 (28.1)	0.010
Asian	524 (10.4)	12,054 (6.6)	0.136	513 (10.2)	524 (10.4)	0.007
Other Race	253 (5.0)	7,261 (4.0)	0.050	252 (5)	242 (4.8)	0.009
Unknown Race	528 (10.5)	26,408 (14.4)	0.120	526 (10.5)	491 (9.8)	0.023
Left ventricular ejection fraction, %	47.4 (16.4)	52.6 (15.1)	0.326	47.5 (16.4)	47.9 (17.5)	0.023
Comorbidities, *n* (%)						
Diabetes mellitus	3,891 (77.0)	77,259 (42.2)	0.759	3,856 (76.9)	3,918 (78.1)	0.030
Metabolic disorders	4,489 (88.9)	93,184 (50.9)	0.909	4,453 (88.8)	4,514 (90.0)	0.039
Hypertension	3,827 (75.8)	87,039 (47.5)	0.607	3,794 (75.6)	3,842 (76.6)	0.022
Chronic obstruction pulmonary disease	1,137 (22.5)	20,146 (11.0)	0.313	1,129 (22.5)	1,139 (22.7)	0.005
Cancer	1,344 (26.6)	28,022 (15.3)	0.280	1,334 (26.6)	1,304 (26.0)	0.013
Stroke	510 (10.1)	8,791 (4.8)	0.202	502 (10.0)	466 (9.3)	0.022
Atrial fibrillation	2,061 (40.8)	30,769 (16.8)	0.551	2,026 (40.4)	2,001 (39.9)	0.010
Dementia	147 (2.9)	3,846 (2.1)	0.050	145 (2.9)	130 (2.6)	0.018
Medications, *n* (%)						
ACEis	956 (18.9)	23,527 (12.8)	0.167	953 (19.0)	971 (19.4)	0.009
ARBs	2,336 (46.2)	25,765 (14.1)	0.749	2,300 (45.9)	2,261 (45.1)	0.016
Beta blockers	4,016 (79.5)	82,248 (44.9)	0.763	3,980 (79.3)	4,076 (81.3)	0.048
Alpha blockers	1,276 (25.3)	21,176 (11.6)	0.359	1,262 (25.2)	1,269 (25.3)	0.003
Calcium channel blockers	2,773 (54.9)	64,160 (35)	0.407	2,758 (55.0)	2,835 (56.5)	0.031
Furosemide	3,436 (68.0)	62,454 (34.1)	0.719	3,396 (67.7)	3,501 (69.8)	0.045
Spironolactone	1,226 (24.3)	9,785 (5.3)	0.553	1,197 (23.9)	1,131 (22.5)	0.031
Entresto	867 (17.2)	2,045 (1.1)	0.580	831 (16.6)	782 (15.6)	0.027
Ivabradine	53 (1.0)	311 (0.2)	0.113	51 (1.0)	46 (0.9)	0.010
Digitalis glycosides	263 (5.2)	3,088 (1.7)	0.194	258 (5.1)	258 (5.1)	0.000
Vericiguat	13 (0.3)	16 (0.0)	0.068	10 (0.2)	10 (0.2)	0.000
Organic nitrates	1,990 (39.4)	34,089 (18.6)	0.470	1,968 (39.2)	1,972 (39.3)	0.002
Platelet aggregation inhibitors	3,132 (62.0)	58,717 (32.1)	0.629	3,101 (61.8)	3,147 (62.7)	0.019
HMG CoA reductase inhibitors	3,671 (72.7)	64,669 (35.3)	0.808	3,637 (72.5)	3,749 (74.7)	0.051
Ezetimibe	406 (8.0)	3,621 (2.0)	0.281	389 (7.8)	352 (7.0)	0.028
Evolocumab	66 (1.3)	281 (0.2)	0.136	60 (1.2)	60 (1.2)	0.000
Alirocumab	23 (0.5)	120 (0.1)	0.077	19 (0.4)	24 (0.5)	0.015

ACEi, angiotensin-converting enzyme inhibitor; ARB, angiotensin receptor blocker; ARNI, angiotensin receptor-neprilysin inhibitor; SD, standard deviation; SGLT2i, sodium-glucose cotransporter-2 inhibitor.

### Primary outcome

**Figure 2:**
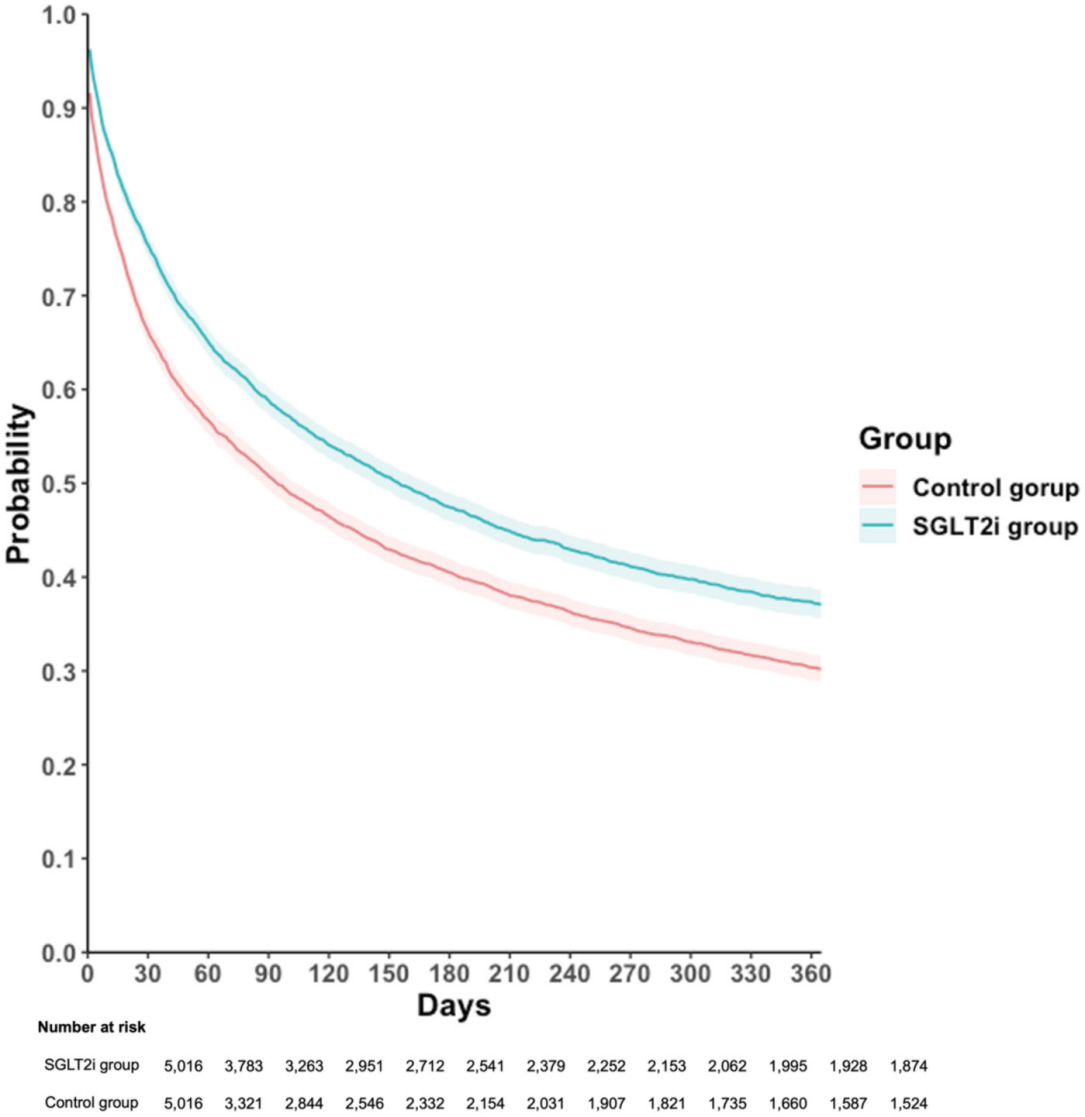
Kaplan–Meier time-to-event free curves of the composite outcome comparison to SGLT2i and control groups. SGLT2i, sodium-glucose co-transporter 2 inhibitor. Primary outcome was defined as composite event of all-cause hospitalization, all-cause mortality, and acute pulmonary edema.

For the primary outcome, which included all-cause hospitalization, all-cause mortality, and acute pulmonary edema, was observed in 2,696 patients in the SGLT2i group, corresponding to an incidence rate of 65.22 per 100 person-years, compared to 3,227 events in the control group with an incidence rate of 64.38 per 100 person-years. In crude analyses, the observed incidence rates appeared similar between groups. However, the Cox proportional hazards model, which accounts for censoring and the timing of events, demonstrated that SGLT2i users had a significantly lower risk of the primary composite outcome compared with non-users (HR, 0.80; 95% CI, 0.76–0.84; *p* < 0.001), with an E-value of 1.61 [95% confidence limit (LCL), 1.51; Table 2]. Kaplan–Meier curves consistently demonstrated a higher event-free probability in the SGLT2i group than the control group (log-rank *p* < 0.001, Figure 2).

**Table 2:** Hazard ratio of outcomes between SGLT2i and control groups.

Outcome	SGLT2i group (*n* = 5,016)		Control group (*n* = 5,016)		HR (95% CI)	*P* value	*E*-value (95% LCL)
	Events (*n*)	Incidence rate per 100 person-years	Events (*n*)	Incidence rate per 100 person-years			
Primary outcome							
Composite outcome	2,696	65.22	3,227	64.38	0.80 (0.76,0.84)	<.001	1.61 (1.51)
Secondary outcomes							
All-cause mortality	648	15.68	999	19.93	0.69 (0.63,0.76)	<.001	2.26 (1.96)
All-cause hospitalization	2,403	58.13	2,686	53.59	0.87 (0.82,0.92)	<.001	1.44 (1.31)
Acute pulmonary edema	267	6.46	339	6.76	0.84 (0.71,0.98)	0.027	1.67 (1.53)

CI, confidence interval; HR, hazard ratio; LCL, lower confidence limit.

Primary outcome was defined as composite event of all-cause hospitalization, all-cause mortality, and acute pulmonary edema.

**Figure 3:**
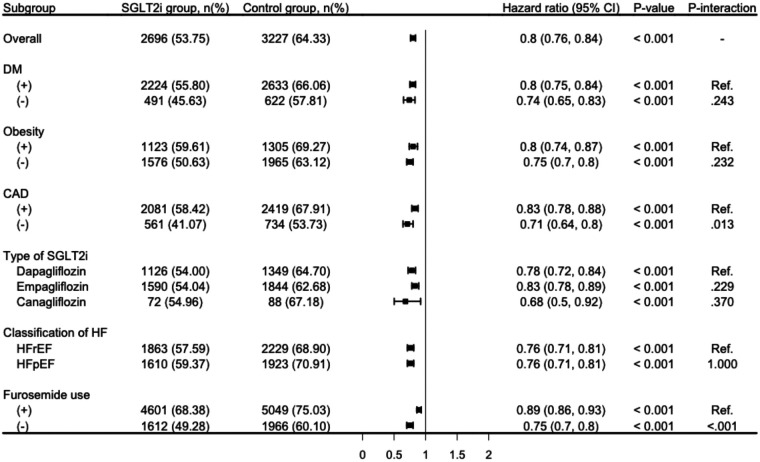
Subgroup analysis for the risk of composite outcome comparison to SGLT2i and control groups. CAD, coronary artery disease; DM; diabetes mellitus; HF, heart failure; HFrEF, heart failure with reduced ejection fraction; HFpEF, heart failure with preserved ejection fraction; SGLT2i, sodium-glucose co-transporter 2 inhibitor primary outcome was defined as composite event of all-cause hospitalization, all-cause mortality, and acute pulmonary edema.

The lower cumulative incidence of primary outcome remained consistent across stratified analyses by diabetes status, obesity, CAD, types of SGLT2i, and heart failure classification. In patients with diabetes, SGLT2i use was associated with a significantly lower risk of the primary outcome (HR, 0.80; 95% CI: 0.75–0.84; *p* < 0.001), with a similar trend observed in those without diabetes (HR, 0.74; 95% CI, 0.65–0.83; *p* < 0.001; Figure 3). The protective effect was also evident in both obese (HR, 0.80; 95% CI, 0.74–0.87; *p* < 0.001) and non-obese individuals (HR, 0.75; 95% CI, 0.70–0.82; *p* < 0.001; Figure 3).

Similarly, patients with CAD experienced a significant reduction in the primary outcome risk with SGLT2i treatment (HR, 0.83; 95% CI, 0.78–0.88; *p* < 0.001), and the effectiveness was consistent in those without CAD (HR, 0.71; 95% CI, 0.64–0.80; *p* < 0.001; Figure 3). When stratified by the type of SGLT2i, all three agents showed protective effectiveness, with dapagliflozin (HR, 0.78; 95% CI, 0.72–0.84; *p* < 0.001), empagliflozin (HR, 0.83; 95% CI, 0.78–0.89; *p* < 0.001), and canagliflozin (HR, 0.68; 95% CI, 0.50–0.92; *p* = 0.001; Figure 3) all demonstrating statistically significant risk reductions. Furthermore, the benefit of SGLT2i use was observed in both heart failure with reduced ejection fraction (HFrEF) (HR, 0.76; 95% CI, 0.71–0.81; *p* < 0.001) and heart failure with preserved ejection fraction (HFpEF) (HR, 0.76; 95% CI, 0.71–0.81; *p* < 0.001; Figure 3). Consistent results were also observed after stratifying by baseline furosemide use, with SGLT2i associated with lower risk of the primary outcome in both furosemide users (HR, 0.89; 95% CI, 0.86–0.93) and non-users (HR, 0.75; 95% CI, 0.70–0.80; both *p* < 0.001; Figure 3).

### Secondary outcomes

Among the secondary outcomes, the SGLT2i group demonstrated a significantly lower risk of all-cause mortality compared to the control group. A total of 648 deaths occurred in the SGLT2i group, with an incidence rate of 15.68 per 100 person-years, whereas 999 deaths were recorded in the control group, corresponding to an incidence rate of 19.93 per 100 person-years. This resulted in an HR of 0.69 (95% CI, 0.63–0.76; *p* < 0.001). The E-value for this outcome was 2.26 (95% LCL, 1.96; Table 2).

In terms of all-cause hospitalization, the incidence rate was 58.13 per 100 person-years in the SGLT2i group compared to 53.59 per 100 person-years in the control group. The SGLT2i group had 2,403 hospitalization events, while the control group had 2,686 events. The HR was 0.87 (95% CI, 0.82–0.92; *p* < 0.001) with the corresponding E-value of 1.44 (95% LCL, 1.31).

Furthermore, the incidence of acute pulmonary edema was lower in the SGLT2i group, with 267 events and an incidence rate of 6.46 per 100 person-years, compared to 339 events in the control group with an incidence rate of 6.76 per 100 person-years. The HR for this outcome was 0.84 (95% CI, 0.71–0.98; *p* = 0.027) with the corresponding E-value was 1.67 (95% LCL, 1.53). We compared the incidence of syncope and diabetic ketoacidosis between SGLT2i and DPP4i users (Supplementary Table S9). The risk of syncope was not significantly different between groups (HR 0.997, 95% CI 0.899–1.105, *p* = 0.950). In contrast, SGLT2i use was associated with a modestly increased risk of diabetic ketoacidosis (HR 1.328, 95% CI 1.029–1.716, *p* = 0.029).

### Sensitivity analysis

Associations between SGLT2i use and the two negative control outcomes: traumatic brain injury (HR, 0.84; 95% CI, 0.56–1.25) and skin cancer (HR, 1.37; 95% CI, 0.98–1.90) were not statistically significant (Supplementary Table S4). The landmark analysis in Supplementary Table S5 evaluated outcomes across three distinct time periods. Regarding the primary outcome, the SGLT2i group showed lower risks in the 1-month to 1-year analysis (HR, 0.92; 95% CI, 0.87–0.97; *P* = 0.004), 2-month to 1-year analysis (HR, 0.89; 95% CI, 0.84–0.95; *P* < 0.001), and 3-month to 1-year analysis (HR, 0.85; 95% CI, 0.79–0.91; *P* < 0.001) (Supplementary Table S5). Furthermore, varying the time window for SGLT2i initiation within six months after diagnosis demonstrated consistent outcomes (HR 0.88, 95% CI 0.83–0.92; *P* < 0.001) (Supplementary Table S6). The comparison between the SGLT2i group and the DPP4is group showed concordant outcomes (HR, 0.91; 95% CI, 0.86–0.97; *P* = 0.002) (Supplementary Table S7). The analysis, which was restricted to patients in the SGLT2i group with a second prescription recorded between 6 months and 1 year after the index date, demonstrated consistent outcomes, further confirming the reliability of our results (HR, 0.89; 95% CI, 0.81–0.96; *P* < 0.001) (Supplementary Table S8).

## Discussion

This exploratory study, which included 10,032 patients, revealed that in those with ESKD and HF, SGLT2 inhibitors were significantly associated with lower one-year composite outcomes of all-cause hospitalization, all-cause mortality, or acute pulmonary edema. For secondary outcomes, SGLT2i use was also linked to reduced cumulative incidences of these individual events. Subgroup analyses demonstrated consistent results across various strata, including HFrEF, HFpEF, the presence or absence of T2D, CAD, obesity, and different SGLT2i (dapagliflozin, empagliflozin, and canagliflozin). Negative control analysis using traumatic brain injury and skin cancer showed no significant differences between groups. Additionally, landmark analyses were conducted using different follow-up periods, specifically from 1-month to 1-year, 2-month to 1-year analysis, and 3-month to 1-year to evaluate whether prognosis varied. The results remained consistent for the composite outcomes. The real-world nature of this cohort study provides clinically relevant insights into the effectiveness of SGLT2is in patients with HF and ESKD, a population largely excluded from randomized trials. By capturing routine prescribing patterns, polypharmacy, and heterogeneous disease severity, our findings complement existing trial evidence and help inform treatment considerations in daily clinical practice.

CKD is common among patients with HF, which is a major contributor to hospitalization, morbidity, and mortality (16). Our findings align with those reported in large randomized trials of patients with advanced CKD not requiring dialysis. In the DAPA-CKD trial, dapagliflozin reduced the composite of sustained eGFR decline, ESKD, or death from renal or cardiovascular causes, and also lowered the risk of cardiovascular death or hospitalization for heart failure (17). Similarly, the CREDENCE trial demonstrated that canagliflozin reduced cardiovascular events in patients with diabetic CKD (18). Notably, the magnitude of risk reduction in these trials is remarkably similar to that observed in our real-world ESKD cohort. Together, these findings suggest that the cardiovascular benefits of SGLT2i may persist even in the dialysis population, despite their exclusion from prior randomized studies.

A meta-analysis including 12,251 patients demonstrated that SGLT2i significantly reduces the risk of cardiovascular death and heart failure hospitalizations across a broad range of ejection fraction in heart failure patients (19). Another prespecified analysis of the EMPEROR-Reduced trial, which included 1,978 patients with HF and CKD, further revealed that empagliflozin significantly reduced the cardiovascular mortality or HF hospitalizations (HR 0.78, 95% CI, 0.65–0.93) (20). Moreover, a systematic review and meta-analysis of 13 trials involving 90,402 participants demonstrated that SGLT2i reduce the risk of renal and HF outcomes across all eGFR categories, with the greatest risk reduction for HF observed in individuals with lower eGFR values (11). These findings suggest the potential benefits of SGLT2i in patients with HF and ESKD.

Unfortunately, there is currently a lack of trial-level and observational evidence on the use and effectiveness of SGLT2i in patients with HF who are on dialysis. Our study addresses this existing evidence gap by demonstrating the potential benefits of SGLT2 inhibitors in patients with HF and ESKD.

Urinary glucose excretion induced by SGLT2i decreases linearly as kidney function declines and becomes negligible when eGFR falls below 30 mL/min/1.73 m^2^ (21). Therefore, the benefits of SGLT2i in patients with HF and ESKD are unlikely to be attributed to glycosuria, suggesting alternative mechanisms might be involved. Several key pathophysiological mechanisms in patients with HF and ESKD may explain the benefits of SGLT2 inhibitors, including oxidative stress and inflammation, autophagy regulation, and iron utilization (22).

In patients with HF and ESKD, The autophagy-lysosome system plays a crucial role in cellular homeostasis by capturing misfolded proteins, damaged organelles, and pathogens in autophagosomes for degradation by lysosomal proteases (23). This process is tightly regulated by key signaling pathways, including mTOR, AMPK, glycogen synthase kinase 3 beta (GSK-3β), and the Hippo pathway (24). In patients with HF and ESKD, dysregulation of autophagy might play a crucial role in worse pathological outcomes (25). In animal studies, SGLT2i have been shown to modulate cardiac autophagy and lysosomal degradation by restoring AMPK-mTOR-mediated autophagy (26). Additionally, empagliflozin inhibited excessive autophagy activation in murine diabetic cardiomyopathy by suppressing GSK-3β, leading to the reversal of cardiac dysfunction (27). These findings suggest that the anti-inflammatory and antioxidative effects of SGLT2i extend beyond their renal effects (e.g., glucosuria and natriuresis), highlighting their potential therapeutic role in HF and ESKD.

Iron deficiency is also common in both HF and CKD and is associated with adverse outcomes (28). The underlying mechanism involves the downregulation of hypoxia-inducible factor-2*α* (HIF-2*α*), leading to a dysfunctional iron regulatory system and anemia in patients with HF and ESKD (29, 30). Clinical trials have demonstrated that SGLT2i can increase hematocrit levels, reduce the risk of anemia, and decrease the need for iron supplements or erythropoiesis-stimulating agents (31, 32). These effects are attributed to enhanced erythropoietin production and reduced hepcidin levels, mediated by decreased inflammation and the activation of nutrient deprivation signaling pathways, such as sirtuin-1 (33). *in vitro* studies have also demonstrated that SGLT2i may stimulate HIF-2α via sirtuin-1 activation, thereby promoting erythropoietin production in the liver. These findings suggest that SGLT2i may serve as a potential therapeutic option for patients with HF and ESKD. Given the limited evidence in dialysis populations, mechanistic speculation should remain cautious. More importantly, our results are consistent with findings from pivotal randomized trials in CKD patients not requiring dialysis, including DAPA-CKD, CREDENCE, and EMPA-KIDNEY (34), all of which demonstrated significant reductions in cardiovascular and renal outcomes with SGLT2i. Although these trials excluded patients on dialysis, the similarity in effect estimates suggest that the cardiovascular benefits of SGLT2i may extend to this high-risk group. Our real-world findings therefore complement and extend prior evidence, highlighting the need for dedicated prospective trials in the dialysis population.

This study leveraged a large multicenter database and PSM to enhance statistical power and reduce the influence of measured confounders. Our findings indicate that in patients with HF and ESKD, SGLT2i use is significantly associated with a lower one-year risk of the composite outcome, including all-cause hospitalization, all-cause mortality, and acute pulmonary edema. Sensitivity analyses further confirmed the robustness of these results. Future studies are needed to validate these findings and explore the long-term effects of SGLT2i in patients with HF and ESKD.

### Limitations

This study has several limitations. First, because TriNetX is a registry-based platform, misclassification or underrepresentation of diagnoses and outcomes is possible. To mitigate this, we performed negative control analyses that showed no significant associations, suggesting minimal registration bias. Second, information on drug exposure was limited, as TriNetX does not provide detailed prescription duration, dosage, or refill adherence. We therefore restricted follow-up to one year and conducted a sensitivity analysis requiring a second prescription within 6–12 months, which confirmed consistent results. Nevertheless, long-term effects and treatment persistence could not be fully assessed. In addition, although patients commonly received multiple guideline-directed therapies for heart failure (e.g., ARNI/ACEi/ARB, beta-blockers, and mineralocorticoid receptor antagonists), we did not perform outcome analyses stratified by specific medication combinations. Such analyses would require detailed treatment sequencing and interaction modeling, which are not feasible within the aggregated data structure of the TriNetX platform and may lead to unstable estimates in highly stratified subgroups. Third, despite extensive covariate adjustment and propensity score matching, unmeasured confounding cannot be excluded. We used E-values to estimate the strength of confounding required to explain our findings, which supported the robustness of the results. Fourth, because this was a comparison of initiators vs. non-initiators rather than a randomized trial, immortal time and time-varying confounding may have influenced the results. To address this, we prespecified landmark analyses, which yielded consistent outcomes. Fifth, outcomes were defined using ICD-10 codes. Cause-specific mortality and adjudicated heart failure hospitalizations were unavailable, necessitating use of a broader composite endpoint. Similarly, ESRD was identified via ICD-10 N18.6, without consistent procedural coding for dialysis. While most patients with HF and ESRD are dialysis-dependent, we could not always distinguish dialysis from non-dialysis ESKD. Sixth, safety endpoints such as syncope and ketoacidosis were rare and not adjudicated, limiting the precision of risk estimates. Larger datasets with detailed safety monitoring are needed to confirm these findings. Finally, residual urine output could not be assessed because TriNetX lacks urine output data. Prospective studies that capture dialysis modality parameters and measured urine output are needed to test whether effectiveness differs by residual diuresis.

## Conclusion

SGLT2i is significantly associated with a lower one-year incidence of all-cause mortality, all-cause hospitalization, and acute pulmonary edema in patients with HF and ESKD compared to those not receiving SGLT2i. These findings offer valuable insights into the management of HF and ESKD, a dilemma with limited therapeutic options despite its growing prevalence.

This study highlights the value of real-world data in providing a more comprehensive understanding of the clinical utility of SGLT2i in this high-risk population. However, the observational nature of the study emphasizes the need for further clinical trials to validate these findings.

## Data Availability

As this study was conducted using the TriNetX Global Collaborative Network, the underlying individual-level patient data cannot be publicly shared due to data use agreements and patient privacy protections. However, the data are available to qualified researchers through the TriNetX platform. A direct link to the TriNetX research network access page: https://trinetx.com/real-world-data/. Researchers with appropriate institutional access may request access to the dataset through this platform to replicate or extend our analyses.
