# Supplementary material for: Real-world effectiveness of SGLT2 inhibitors in patients with HF and ESKD: a multicenter cohort study
Source: Front Cardiovasc Med. 2026 Jan 16;13:1652863. doi: 10.3389/fcvm.2026.1652863 (PMC12856922; doi:10.3389/fcvm.2026.1652863)

**eTable 1.** Demographic, diagnostic, procedural, medication, visit, and laboratory codes used in the definition of the cohorts

| **Category** | **Code** | **Description** |
| --- | --- | --- |
| **SGLT2i group** | | |
| **#1**: At least 18 years old | | |
| Demographics | Age | Age (at least 18 years) |
| **#2**: Patients with ESRD and HF and SGLT2i.  (# 2.1 must be fulfilled within 1 year before #2.2) | | |
| **#2.1:** Patients with SGLT2i | | |
| medication | NLM:ATC:A10BK | Sodium-glucose co-transporter 2 (SGLT2) inhibitors |
| **#2.2**: Patients with ESRD and HF (have any of the following) | | |
| diagnosis | UMLS:ICD10CM:I50 | Heart failure |
| diagnosis | UMLS:ICD10CM:N18.6 | End stage renal disease |
| **#3**: Visit HCOs since 2016 | | |
| Visit | Visit | Visit more than twice since 2016 |
| **Control group** | | |
| **#1**: At least 18 years old | | |
| Demographics | Age | Age (at least 18 years) |
| **#2**: Patients with ESRD and HF | | |
| diagnosis | UMLS:ICD10CM:I50 | Heart failure |
| diagnosis | UMLS:ICD10CM:N18.6 | End stage renal disease |
| **#3**: **#3**: Without SGLT2i use during the study period. (cannot have any of the following) | | |
| medication | NLM:ATC:A10BK | Sodium-glucose co-transporter 2 (SGLT2) inhibitors |
| **#4**: Visit HCOs since 2016 | | |
| Visit | Visit | Visit more than twice since 2016 |

**eTable 2.** Demographic, diagnostic, and laboratory codes used in the definition of covariates

| **Category** | **Code** | **Description** |
| --- | --- | --- |
| Demographics | AI | Age at index |
| Demographics | F | Female |
| Demographics | 2106-3 | White |
| Demographics | UNK | Unknown Race |
| Demographics | 2054-5 | Black or African American |
| Demographics | 2028-9 | Asian |
| Demographics | 2131-1 | Other Race |
| Diagnosis | I10 | Essential (primary) hypertension |
| Diagnosis | E08-E13 | Diabetes mellitus |
| Diagnosis | E70-E88 | Metabolic disorders |
| Medication | CV050 | DIGITALIS GLYCOSIDES |
| Medication | CV100 | BETA BLOCKERS/RELATED |
| Medication | CV150 | ALPHA BLOCKERS/RELATED |
| Medication | CV200 | CALCIUM CHANNEL BLOCKERS |
| Medication | CV800 | ACE INHIBITORS |
| Medication | CV805 | ANGIOTENSIN II INHIBITOR |
| Medication | 1656328 | sacubitril |
| Medication | 1649480 | ivabradine |
| Medication | 2475830 | vericiguat |
| Medication | 9997 | spironolactone |
| Medication | C01DA | Organic nitrates |
| Medication | C10AA | HMG CoA reductase inhibitors |
| Medication | 341248 | ezetimibe |
| Medication | 1665684 | evolocumab |
| Medication | 1659152 | alirocumab |
| Medication | BL117 | PLATELET AGGREGATION INHIBITORS |

**eTable 3.** Diagnostic, visit, and procedural codes used in the definition of outcomes

| **Category** | **Code** | **Description** |
| --- | --- | --- |
| **#1**: Composite outcome (have any of the following) | | |
| Diagnosis | UMLS:ICD10CM:J81.0 | Acute pulmonary edema |
| Demographics | Deceased | Deceased |
| Diagnosis | UMLS:ICD10CM:R99 | Ill-defined and unknown cause of mortality |
| Visit | UMLS:HL7V3.0:VisitType:IMP | Visit: Inpatient Encounter |
| Visit | UMLS:HL7V3.0:VisitType:SS | Visit: Short Stay |
| Visit | UMLS:HL7V3.0:VisitType:OBSENC | Visit: Observation Encounter |
| Visit | UMLS:HL7V3.0:VisitType:NONAC | Visit: Inpatient Non-acute |
| Visit | UMLS:HL7V3.0:VisitType:ACUTE | Visit: Inpatient Acute |
| **#2**: All-cause mortality (have any of the following) | | |
| Demographics | Deceased | Deceased |
| Diagnosis | UMLS:ICD10CM:R99 | Ill-defined and unknown cause of mortality |
| **#3**: All-cause hospitalization (have any of the following) | | |
| Visit | UMLS:HL7V3.0:VisitType:IMP | Visit: Inpatient Encounter |
| Visit | UMLS:HL7V3.0:VisitType:SS | Visit: Short Stay |
| Visit | UMLS:HL7V3.0:VisitType:OBSENC | Visit: Observation Encounter |
| Visit | UMLS:HL7V3.0:VisitType:NONAC | Visit: Inpatient Non-acute |
| Visit | UMLS:HL7V3.0:VisitType:ACUTE | Visit: Inpatient Acute |
| **#4**: Acute pulmonary edema | | |
| Diagnosis | UMLS:ICD10CM:J81.0 | Acute pulmonary edema |

**eTable 4**. Negative outcomes between the SGLT2i group and the control group.

| Outcome | HR (95% CI) | *P* value |
| --- | --- | --- |
|  |  |  |
| Traumatic brain injury | 0.84 (0.56,1.25) | 0.380 |
| Skin cancer | 1.37 (0.98,1.90) | 0.060 |

**eTable 5**. Sensitivity analysis with Landmark analysis of primary outcome.

| Outcome | HR (95% CI) | *P* value |
| --- | --- | --- |
|  |  |  |
| 1-month to 1-year |  |  |
| Composite outcome | 0.92 (0.87,0.97) | 0.004 |
| 2-month to 1-year |  |  |
| Composite outcome | 0.89 (0.84,0.95) | < 0.001 |
| 3-month to 1-year |  |  |
| Composite outcome | 0.85 (0.79,0.91) | < 0.001 |

**eTable 6**. Sensitivity analysis by varying the time window for SGLT2i initiation.

| Outcome | HR (95% CI) | *P* value |
| --- | --- | --- |
|  |  |  |
| Initiation of SGLT2i use within 6-months after diagnosis | | |
| Composite outcomes | 0.88 (0.83,0.92) | < 0.001 |

**eTable 7.** Sensitivity analysis for primary outcome comparison between the SGLT2i group and the control group.

| Outcome | No. of patients with outcome | | HR (95% CI) | *P* value |
| --- | --- | --- | --- | --- |
|  | SGLT2i group  (n = 4,130) | control group  (n = 4,130) |  |  |
| Composite outcome | 2,222 | 2,480 | 0.91 (0.86,0.97) | 0.002 |

**eTable 8**. Sensitivity analysis in the SGLT2i group restricted to patients with a second prescription between 6-month and 1-year after the index date.

| Outcome | No. of patients with outcome | | HR (95% CI) | *P* value |
| --- | --- | --- | --- | --- |
|  | SGLT2i group  (n = 1,847) | Control group  (n = 1,847) |  |  |
| Composite outcome | 1,039 | 1,108 | 0.89 (0.81,0.96) | < 0.001 |

**eTable 9**. Safety outcome comparison between the SGLT2i group and the control group.

| Outcome | HR (95% CI) | *P* value |
| --- | --- | --- |
|  |  |  |
| Syncope | 0.997 (0.899,1.105) | 0.950 |
| Ketoacidosis | 1.328 (1.029,1.716) | 0.029 |

**eFigure 1.** Covariate Balance Before and After Propensity Score Matching.


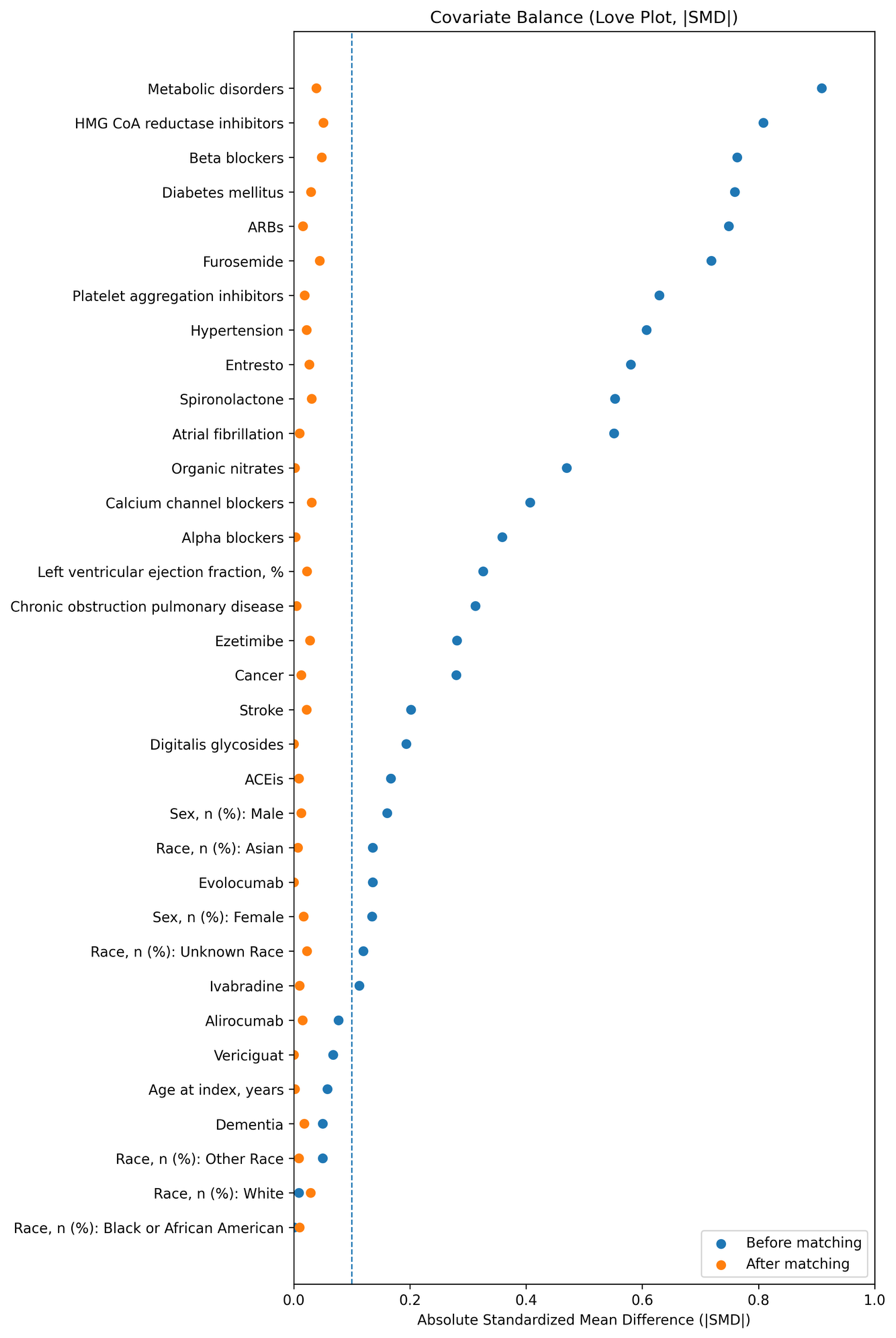

Supplement: Supplementary file 1 [file Datasheet1.docx]
